# Projecting water availability and quality for reuse under scarcity in the Bahr El-Baqar catchment in Egypt using the SIWARE model

**DOI:** 10.1038/s41598-026-49708-4

**Published:** 2026-05-04

**Authors:** Muhammad Ahmad Abdul-Muttalib, Aiman El-Saadi, Hossam El-Gazzar, Mahmoud Ali Refaey

**Affiliations:** 1 Civil Engineering Department, Faculty of Engineering at Shoubra, Benha University, 108 Shoubra St, Cairo, 11629 Egypt; 2 Nile Research Institute, National Water Research Centre, 13621, Delta Barrage, Egypt; 3 Drainage Research Institute, National Water Research Centre, 13621, Delta Barrage, Egypt

**Keywords:** Agricultural drainage water reuse, Water scarcity, SIWARE model, Water quality, Salinity management, Predictive modeling, Nile Delta, Bahr El-Baqar, Climate sciences, Environmental sciences, Hydrology

## Abstract

Agricultural drainage water reuse is a vital strategy for mitigating freshwater scarcity in Egypt. This study applies the SIWARE (Simulation of Water Management in the Arab Republic of Egypt) model to project water quantity and quality under scarcity at two strategic nodes in the Bahr El-Baqar catchment. The first node is the Bahr El-Baqar Feeder (BBF), which supplies the world’s largest wastewater treatment plant. The second node is the Bilad El-Ayad Pump Station (BAP), which supports direct local irrigation. The model was parameterized using extensive data from four Egyptian national authorities. It was rigorously calibrated and validated for 2020–2021. The model achieved strong performance (R² up to 0.92, Nash-Sutcliffe efficiency up to 0.91). Freshwater allocation reductions to the Eastern Delta were simulated at 5% increments from 0% to 50%. Results reveal starkly contrasting node-specific responses. At BBF, baseline total dissolved solids (TDS ~ 2200 ppm) already exceeds Egypt’s 2000 ppm regulatory limit. Closing this gap would require a theoretical increase in dilution, but such an increase is infeasible under Egypt’s fixed 55.5 billion cubic meters (BCM) annual Nile allocation. Under a 50% reduction, TDS at BBF exceeds 3000 ppm (a 36% increase) with a 47% discharge decline. This poses operational risks including accelerated membrane fouling, increased energy consumption, and shortened maintenance cycles. In contrast, BAP shows greater resilience. TDS increases by only 16.4% under the same scenario, remaining below 2000 ppm. However, discharge declines by 45%. Site-specific exponential relationships (R² ≈ 0.98 for curve fitting, 95% confidence intervals ± 4–6%) predict discharge and TDS for any allocation reduction. This enables rapid assessment without full model re-runs. A deployable portfolio of interventions is presented. These include membrane-based pretreatment (up to 82% TDS reduction), temporal abstraction management, upstream salinity source control (75.67% TDS reduction), salt-tolerant crop varieties, and operational blending optimization. The contrasting responses underscore the need for node-specific management. This study provides a quantitative framework and actionable interventions. It supports adaptive water governance and infrastructure planning under increasing scarcity. The work contributes directly to Sustainable Development Goal (SDG) 6 (clean water and sanitation) and SDG 2 (zero hunger).

## Introduction

Water scarcity occurs when available supply cannot meet demand. It also arises when water quality fails to support human and ecosystem functions^1,2^. Causes include climate variability, population growth, urbanization, pollution, and weak governance^3–5^. These factors threaten health, agriculture, economies, and ecosystems^3–5^. In response, many countries are prioritizing effective water management strategies^6–11^.

Egypt, with over 100 million people, receives an annual allocation of 55.5 billion cubic meters (BCM) of water from the Nile River. This allocation is subject to increasing pressures from upstream developments, population growth, and climate variability, which collectively intensify Egypt’s water scarcity challenge^12,13^. The Grand Ethiopian Renaissance Dam (GERD), in particular, has introduced new uncertainties regarding future Nile flows and Egypt’s water security^13^. The country’s current per capita share falls below the World Business Council for Sustainable Development’s benchmark of 1000 m³ per person per year, underscoring a serious scarcity challenge^12^. To bridge this gap, unconventional water sources are vital for irrigation^14–18^. Egypt generates approximately 17 BCM of agricultural drainage water (ADW) annually. Over 75% of this water is discharged into the Mediterranean Sea^19^. Reusing this drainage water for irrigation is therefore considered one of Egypt’s most crucial alternative water resources^20–23^.

Under Egyptian Environmental Law 48/1982, as amended in 2018 by Ministerial Resolution No. 208, the regulatory limit for total dissolved solids (TDS) in drainage water reused for irrigation is 2000 ppm^24^. This limit is the primary salinity standard for drainage water reuse in Egypt, because elevated salinity restricts crop selection, reduces yield, and degrades soil structure over time^10^.

Hydrological and water quality models are essential for assessing ADW reuse feasibility. Globally, such models simulate environmental impacts like groundwater mounding and salination risk to support regulatory decisions^25^. In the Nile Delta, models including QUAL2Kw, MIKE, and SIWARE (Simulation of Water Management in the Arab Republic of Egypt) have evaluated reuse potential under current conditions^26–29^. QUAL2Kw and MIKE have quantified reusable volumes and assessed compliance with water quality standards in specific drains and canals^24,27^. SIWARE has analyzed both formal and informal water reuse. Results show that water shortages drive unofficial reuse, which can restrict crop selection due to elevated salinity^12,30^. More recently, Abdul-Muttalib et al. (2025) applied SIWARE to the Eastern Nile Delta using defined water reduction scenarios^31^. Their work found that system responses remain stable under moderate scarcity but deteriorate sharply under severe reductions. This deterioration includes marked increases in soil salinity and altered patterns of unofficial reuse.

Shaban (2020) analyzed drainage records since 1984, confirming that both reuse volumes and salinity are increasing in the Eastern Nile Delta^32^. This trend reflects the resource’s dynamic and increasingly pressurized state. Historical trend analyses and regional-scale assessments provide valuable insights into reuse dynamics and agricultural impacts. However, their spatial resolution is limited. As a result, they cannot fully capture how water scarcity propagates through the drainage network or manifests at specific reuse control points.

Existing modeling studies demonstrate the utility of hydrological models for diagnosing past and current conditions. However, their focus has been largely retrospective or regional. This predictive gap is especially salient for the Bahr El-Baqar catchment, a region of high strategic importance that now hosts a new mega-treatment facility with a capacity of approximately 5.6 × 10⁶ m³/day^18^. Within this catchment, two primary reuse nodes represent distinct management pathways: the Bahr El-Baqar Feeder (BBF), which supplies the world’s largest treatment plant, and the Bilad El-Ayad Pump Station (BAP), which supports direct local irrigation. Observed measurements indicate that BBF already faces salinity challenges. In contrast, BAP, based on field data, operates within compliance. Evaluating both nodes’ responses to allocation cuts is therefore a pressing management priority.

To address this gap, the present study uniquely applies the SIWARE model to generate quantitative, scenario-based projections of future water quantity and quality at BBF and BAP. Freshwater allocation reductions are simulated from 0% to 50% in 5% increments. The study is guided by three specific questions: (i) how simulated reductions in the Eastern Delta’s water quota affect discharge and TDS at BBF and BAP; (ii) the extent to which projected TDS levels comply with Egyptian regulatory standards under each scenario; and (iii) whether robust empirical relationships can be derived to predict discharge and salinity based on allocation reductions. This approach delivers actionable insights into adaptive water resource management at these critical control points.

## Methodology

### Study area

**Fig. 1:**
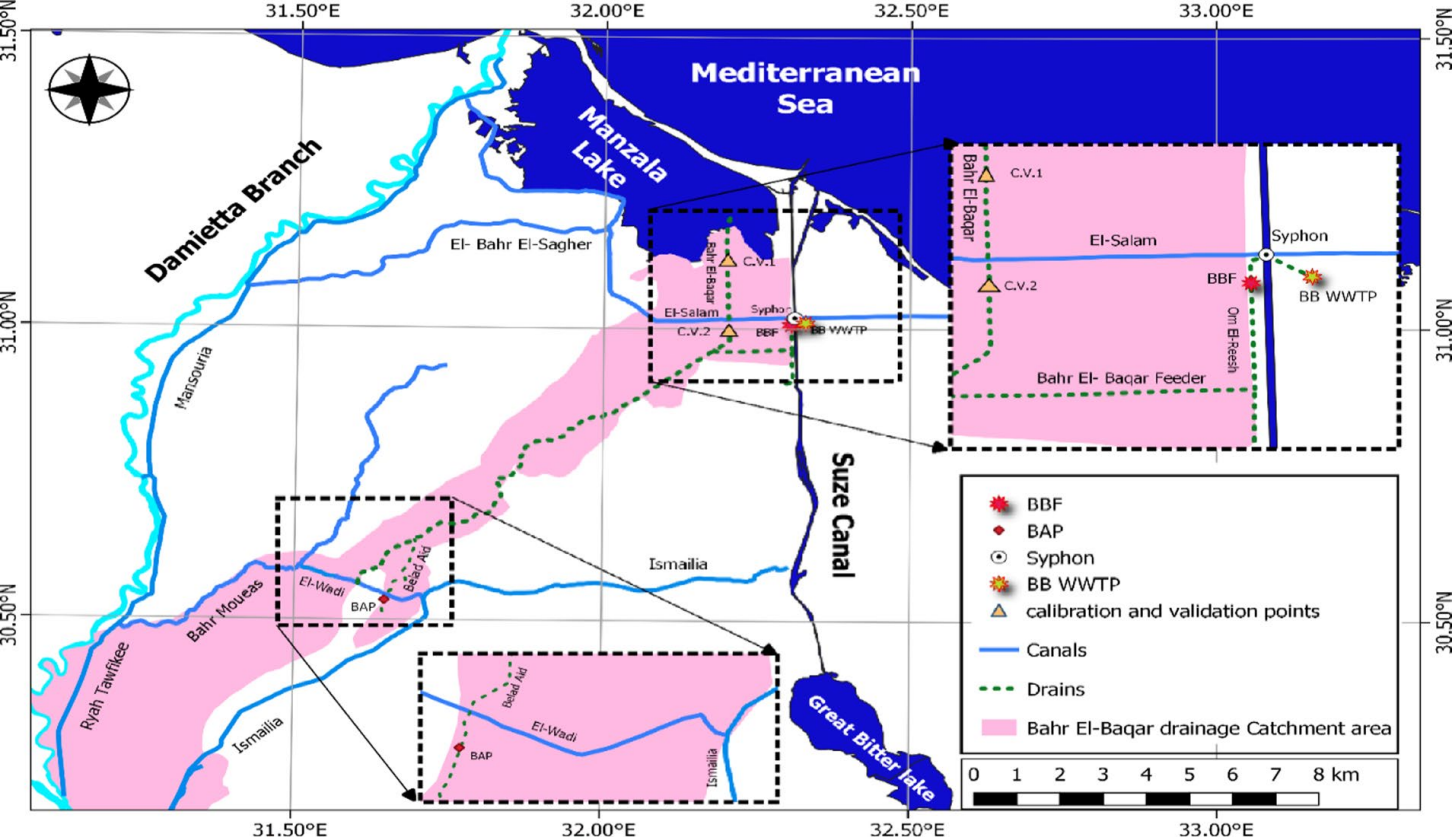
Map of the Bahr El-Baqar catchment in the Eastern Nile Delta, Egypt, showing the primary study sites (BBF, BAP), calibration/validation points (C.V.1, C.V.2), and major hydrologic features including the Damietta Branch of the Nile, the Mediterranean Sea, Lake Manzala, and the Suez Canal. The map was generated using QGIS (version 3.4.13; https://qgis.org) based on spatial datasets obtained from national authorities.

This study focuses on the Bahr El-Baqar catchment in the Eastern Nile Delta, Egypt, a region defined by major hydrologic features including the Damietta Branch of the Nile, the Mediterranean Sea, Lake Manzala, and the Suez Canal, as shown in Fig. 1. All maps were produced using Quantum Geographic Information System (QGIS) software. Within this catchment, two strategic reuse nodes introduced earlier are examined: the Bahr El-Baqar Feeder (BBF) and the Bilad El-Ayad Pump Station (BAP).

The BBF site (32.30°E, 31.02°N) collects drainage water from the Bahr El-Baqar drain after its convergence with the Om El-Reesh drain. Flow is conveyed via a siphon beneath the Suez Canal to the Bahr El-Baqar Wastewater Treatment Plant (BB WWTP), the world’s largest facility of its kind. The BAP site (31.65°E, 30.54°N) abstracts water from the Bilad El-Ayad drain, which is then blended with fresh water from the Wadi Canal for direct irrigation supply.

Model calibration and validation were performed at two independent locations: the South Port Said Pump Station (C.V.1) and the South Al-Hussainiya Plain Pump Station (C.V.2). These sites provide spatially distinct observational datasets for assessing model performance across contrasting hydrological settings within the Eastern Nile Delta.

### SIWARE model setup and configuration

The SIWARE model was used to simulate water quantity and quality in the study area. This integrated deterministic model was developed specifically for the Nile Delta^33^.

SIWARE was selected for two reasons aligned with this study’s objectives. First, it simulates both formal and informal ADW reuse. Second, it couples water quantity with salinity, enabling holistic assessment of reuse viability under scarcity.

**Fig. 2:**
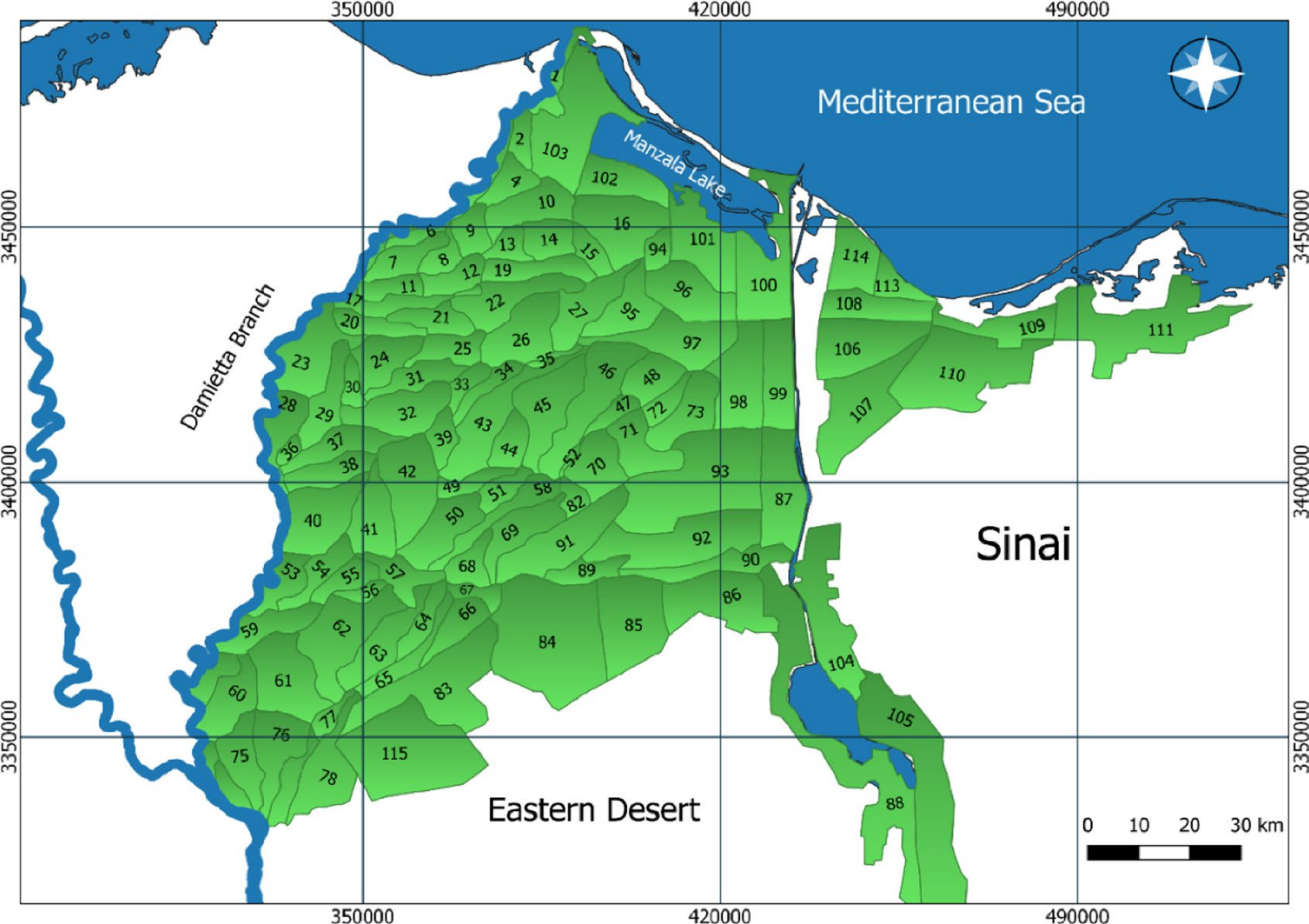
Eastern Delta calculation units (CUs). The spatial delineation and visualization of CUs were performed using QGIS (version 3.4.13; https://qgis.org), integrating boundary data and hydrological network layers.

The study area was subdivided into 115 hydrologically independent calculation units (CUs), each linked to a canal node and drain segment, as shown in Fig. 2. Data sources for parameterization are detailed in Sect.  2.3. This includes soil properties, crop parameters, and irrigation schedules.

When freshwater is insufficient, farmers informally supplement irrigation with nearby drainage water. Regulated reuse occurs at official pumping stations (BBF, BAP), where drainage water is mixed with freshwater at prescribed ratios complying with the Ministry of Water Resources and Irrigation (MWRI) quality standards. However, there are no fixed numerical mixing ratios at these stations. Instead, mixing is determined by compliance with the 2000 ppm TDS limit under Egyptian Law 48/1982 (amended 2018). At each time step, the allowable mixing ratio is calculated as the maximum proportion of drainage water that can be blended with available freshwater so that the resulting TDS ≤ 2000 ppm. This compliance‑based approach applies equally under baseline conditions and under all scarcity scenarios, reflecting actual operational practice in Egypt.

SIWARE operates through four interconnected sub-models^33^. The Design module defines irrigation network structure and control rules. The Water Duty module calculates crop water requirements using soil, crop, and climatic data. The Water Distribution module allocates canal water to CUs following official MWRI schedules. The Reuse module manages both formal and informal reuse: freshwater deficits are automatically supplemented with drainage water, representing farmer-led reuse, while formal reuse at BBF and BAP follows regulated mixing ratios.

### Data sources and model inputs

Model input data were obtained from four Egyptian national authorities, ensuring regional specificity and regulatory relevance. All spatial datasets were processed using QGIS and integrated with the 115 CUs described in Sect.  2.2. Vector layers, including canal networks, drainage systems, and CU boundaries, were incorporated within a unified geospatial framework, and spatial operations such as overlay and intersection were applied. Thematic maps were subsequently generated using graduated symbology to represent spatial variability across the study area.

**Fig. 3:**
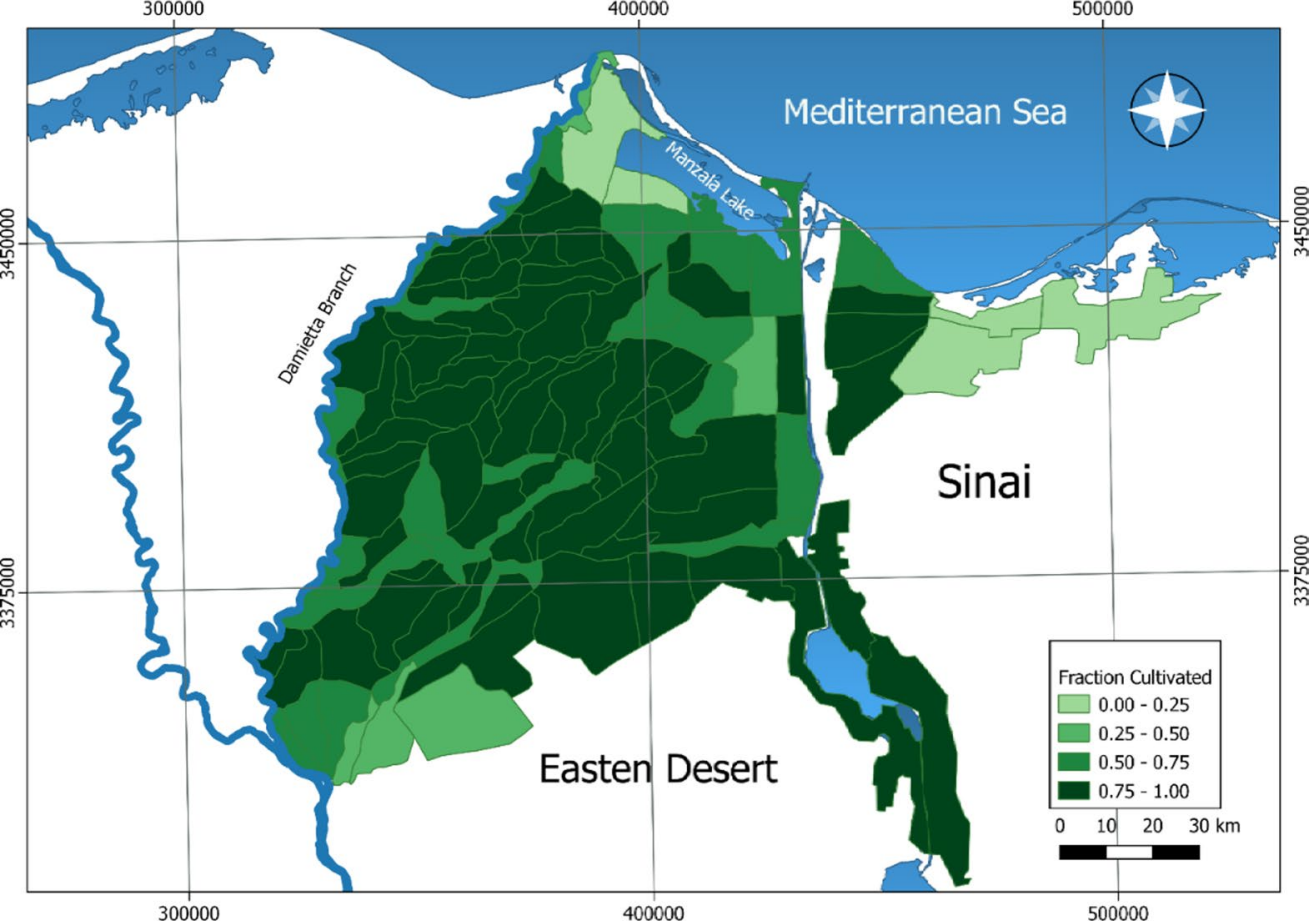
Spatial distribution of cultivated land proportions across calculation units (CUs) in the Eastern Nile Delta for the year 2020. Thematic mapping was conducted using QGIS (version 3.4.13; https://qgis.org) by intersecting crop distribution datasets with CU boundaries and applying graduated color classification.

The Ministry of Agriculture and Land Reclamation (MALR) provided CU boundaries and soil parameters: dry bulk density, moisture content at saturation and wilting point, and diffusivity. MALR also supplied crop distribution maps and cultivated land fractions for each Marakez. These data were spatially intersected with CUs to calculate cultivated area percentages per CU for the year 2020, as demonstrated in Fig. 3. The model represents 15 major crop groups, consolidated based on shared traits including irrigation salinity tolerance, water requirements, plant height, soil coverage, allowable ponding, and root depth. Corresponding water duty values were obtained from MALR for each crop group.

MWRI provided canal network layouts and official water allocation schedules, structured into 36 ten-day periods per year. These define the freshwater supply available to each CU under baseline conditions.

The Ground Water Research Institute (GWRI) supplied aquifer properties including thickness, permeability, clay layer composition, and salinity, along with piezometric level records. These data were used to simulate subsurface flow into CUs, capturing groundwater-surface water interactions.

Long-term meteorological records (10-year averages) were obtained for the Eastern Delta, including rainfall and evaporation. Additional parameters such as solar radiation, mean temperature, humidity, sunshine duration, wind speed, and precipitation were processed to estimate maximum evapotranspiration demand and compute the reduction coefficient for partial soil cover using the Rijtema method^34^.

Finally, the Drainage Research Institute (DRI) provided subsurface drainage design parameters (drain spacing and depth) and observed discharge and salinity records for 2020–2021. These records were used for model calibration and validation at sites C.V.1 and C.V.2.

### Total dissolved solids modeling methodology

Salinity was simulated as TDS in parts per million (ppm). Electrical conductivity (EC) measurements, expressed in deciSiemens per metre (dS/m), were converted to TDS using the standard conversion factor for Nile Delta water: TDS (ppm) = 640 × EC (dS/m)^34,35^. This conversion ensures consistency with regulatory standards (Law 48/1982, 2000 ppm limit) and aligns with salinity monitoring practices in Egyptian water management. Within each CU, the model tracks salt mass through a mass balance approach. Total salt inflow is computed from the TDS concentration and volume of all applied water sources, including canal water and any reused drainage water. The model simulates salt removal through leaching. It uses a calibrated soil leaching fraction for each CU. The TDS concentration of drainage water leaving a CU is then calculated as the ratio of cumulative salt load to total drainage volume. This mass balance approach maintains salt continuity through the system. It also allows salinity to evolve dynamically as water allocations and reuse practices change.

### Modeling experiment design

The experiment assessed how freshwater allocation reductions affect water quantity and quality at BBF and BAP. The experiment followed a systematic step-by-step procedure:

Step 1: Baseline simulation. The calibrated SIWARE model was run using observed 2020–2021 data to establish baseline conditions for discharge and salinity at all calculation units. Model performance was verified against observed data at calibration sites C.V.1 and C.V.2 (Sect.  3.3).

Step 2: Scenario development. Freshwater allocation reductions were simulated at 5% increments from 0% to 50%, resulting in 11 scenarios. The 0% scenario represents current baseline conditions, while the 50% scenario represents an extreme but plausible scarcity condition that could result from upstream developments, population growth, or climate-induced variability in Nile flows.

Step 3: Scenario simulation. For each reduction scenario, freshwater input to every calculation unit was reduced uniformly by the specified percentage across all canals and all 36 ten-day periods. The reduction was applied proportionally to the baseline allocation for each period, maintaining the original seasonal distribution pattern (higher allocations in summer, lower in winter). This approach isolates the effect of total annual allocation reduction while preserving the temporal dynamics of water delivery. All other model parameters, including soil properties, crop distribution, groundwater interactions, and climatic data, were held constant at baseline values to isolate the effect of allocation reductions.

Step 4: Output extraction. Simulated daily discharge and TDS were extracted for the two target nodes (BBF and BAP). Annual average values were calculated for each scenario to characterize the relationship between allocation reduction and both water quantity and quality.

Step 5: Derivation of predictive relationships. Regression analysis was applied to quantify the relationship between allocation reduction percentage and both discharge and TDS at each node. The resulting empirical equations are presented in Sect.  4.1.

Step 6: Uncertainty assessment. Confidence intervals were calculated to quantify the propagation of parameter and calibration uncertainty through the predictive relationships. These intervals are reported alongside the equations in Sect.  4.1.

## Model calibration and validation

### Calibration procedure

The SIWARE model was calibrated for the reference year 2020 using an iterative, manual trial-and-error process. The main parameters governing the water and salt balance, such as irrigation water TDS, field drainage coefficients, canal conveyance efficiencies, and soil leaching fractions, were adjusted within physically plausible ranges. The objective was to minimize the difference between simulated and observed monthly discharge and salinity at sites C.V.1 and C.V.2.

Model performance during calibration was evaluated using the Monthly Average Deviation (Av._dev_.), calculated according to Eq. (1), in addition to standard statistical indicators including the coefficient of determination (R²), root mean square error (RMSE), and Nash Sutcliffe efficiency (NSE), defined in Eq. (2) through (4):

1 $$\:\mathrm{Av.}\mathrm{dev.}=100\times\:\frac{{\sum\:}_{i=1}^{\:\:n}\:\left|{P}_{m,i}-{P}_{c,i}\right|}{{\sum\:}_{i=1}^{\:\:n}\left(\:\frac{{\:P}_{m,i}+{P}_{c,i}}{2}\right)}$$

2 $$\:{R}^{2}=\frac{{\left[{\sum\:}_{i=1}^{n}\left({P}_{m,i}-{\stackrel{-}{P}}_{m}\right)\left({P}_{c,i}-{\stackrel{-}{P}}_{c}\right)\right]}^{2}}{\left[{{\sum\:}_{i=1}^{n}\:\left({P}_{m,i}-{\stackrel{-}{P}}_{m}\right)}^{2}\right]\:\left[{{\sum\:}_{i=1}^{n}\:\left({P}_{c,i}-{\stackrel{-}{P}}_{c}\right)}^{2}\right]}$$

3 $$\:RMSE=\sqrt{{\sum\:}_{i=1}^{n}\frac{{\left({P}_{m,i}-{P}_{c,i}\right)}^{2}}{n}}$$

4 $$\:NSE=1-\:\:\frac{{\sum\:}_{i=1}^{n}{\left({P}_{m,i}-{P}_{c,i}\right)}^{2}}{{\sum\:}_{i=1}^{n}{\:\left({P}_{m,i}-{\stackrel{-}{P}}_{m}\right)}^{2}}$$

Where P_m, i_ and P_c, i_ represent the observed and simulated values, respectively, for month i. These values correspond to discharge (m³/s) and salinity (EC in dS/m). P̄_m_ and P̄_c_ denote their mean values, and *n* = 12 (the number of monthly observations per year). Acceptance thresholds for Av._dev_. (≤ 30% for discharge and ≤ 50% for salinity) followed standards established by the national Drainage Water Reuse Project Steering Committee, representing DRI, MWRI, and the Egyptian government [33–35]. Calibration was repeated until all metrics met these criteria.

### Validation procedure

The calibrated parameter set was fixed and applied without further adjustment to simulate the independent validation year 2021. Model robustness and predictive skill were assessed using the same metrics (Av._dev_., R², RMSE, NSE) at both the primary calibration site (C.V.1) and the independent validation site (C.V.2).

### Results of calibration and validation

**Fig. 4:**
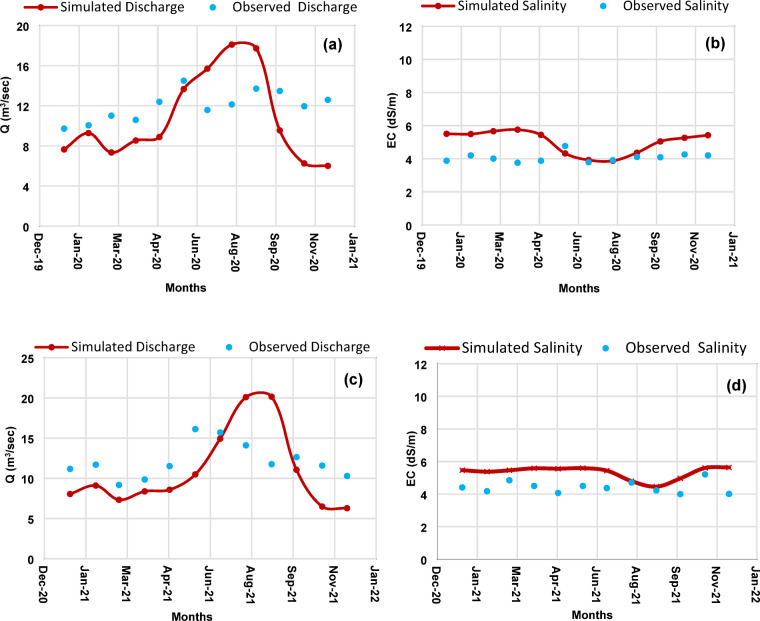
Comparison of simulated and observed monthly mean values at C.V.1: (**a**) discharge (m³/s) in 2020, (**b**) salinity (dS/m) in 2020, (**c**) discharge (m³/s) in 2021, and (**d**) salinity (dS/m) in 2021.

The statistical performance indicators for C.V.1 and C.V.2 are summarized in Tables 1 and 2, respectively. At C.V.1, R² values ranged from 0.81 to 0.90, RMSE ranged from 1.65 to 1.72 m³/s for discharge and from 0.43 to 0.47 dS/m for salinity, while NSE values ranged from 0.78 to 0.88. Corresponding Av._dev_. values remained below 12% for discharge and 21% for salinity, well within the acceptable thresholds, as illustrated in Table 1. Visual comparison of simulated and observed monthly time series at this site is provided in Fig. 4, demonstrating strong agreement in both magnitude and seasonal pattern.

**Table 1:** Statistical performance indicators for calibration and validation at C.V.1.

Variable	Year	*R*²	RMSE	NSE	Av._dev_. (%)
Discharge	2020	0.88	1.72 m³/s	0.86	11.03
Salinity	2020	0.81	0.47 dS/m	0.78	20.59
Discharge	2021	0.90	1.65 m³/s	0.88	10.62
Salinity	2021	0.84	0.43 dS/m	0.82	18.63

**Fig. 5:**
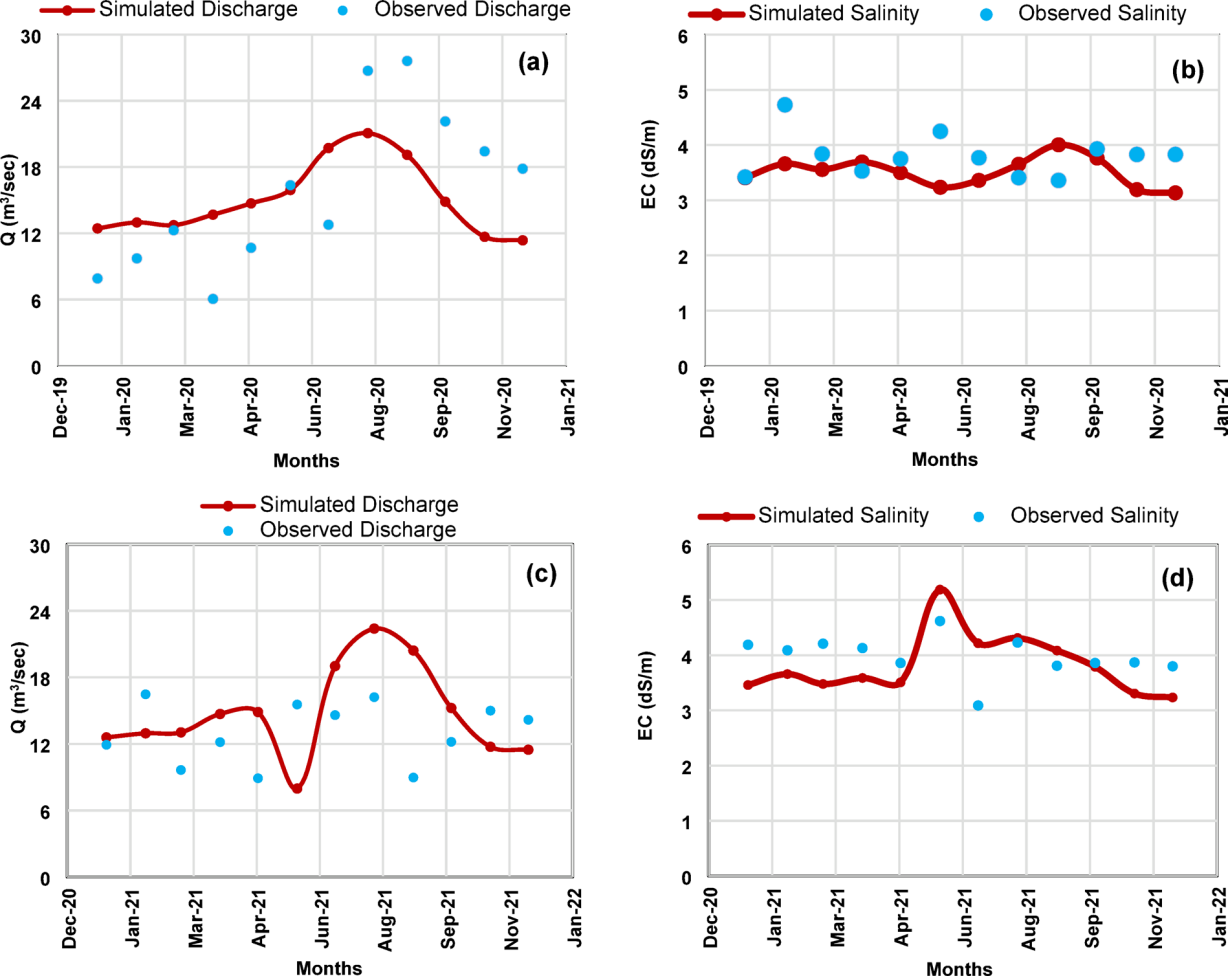
Comparison of simulated and observed monthly mean values at C.V.2: (**a**) discharge (m³/s) in 2020, (**b**) salinity (dS/m) in 2020, (**c**) discharge (m³/s) in 2021, and (**d**) salinity (dS/m) in 2021.

Performance at C.V.2 was similarly robust. R² values ranged from 0.83 to 0.92, RMSE from 1.38 to 1.54 m³/s for discharge and 0.31 to 0.39 dS/m for salinity, and NSE from 0.80 to 0.91. Av._dev_. values remained consistently low, well below prescribed limits, as demonstrated in Table 2. The corresponding time series comparison at C.V.2 (Fig. 5) confirms the model’s ability to capture interannual variability and site-specific hydrological responses.

Overall, these results demonstrate that SIWARE accurately reproduces both the magnitude and seasonal variability of discharge and salinity across sites and years. Examination of the monthly time series during 2020–2021 revealed no extreme salinity spikes exceeding 2 standard deviations from the mean, indicating that the calibration period captures typical operational conditions. This strong performance establishes confidence in the model’s suitability for subsequent scenario analyses.

**Table 2:** Performance evaluation metrics for discharge and salinity at C.V.2.

Variable	Year	*R*²	RMSE	NSE	Av._dev_. (%)
Discharge	2020	0.92	1.38 m³/s	0.91	4.80
Salinity	2020	0.86	0.31 dS/m	0.84	7.20
Discharge	2021	0.89	1.54 m³/s	0.87	12.40
Salinity	2021	0.83	0.39 dS/m	0.80	4.11

## Results and discussion

### Effects of eastern delta water allocation reductions on reuse water

**Fig. 6:**
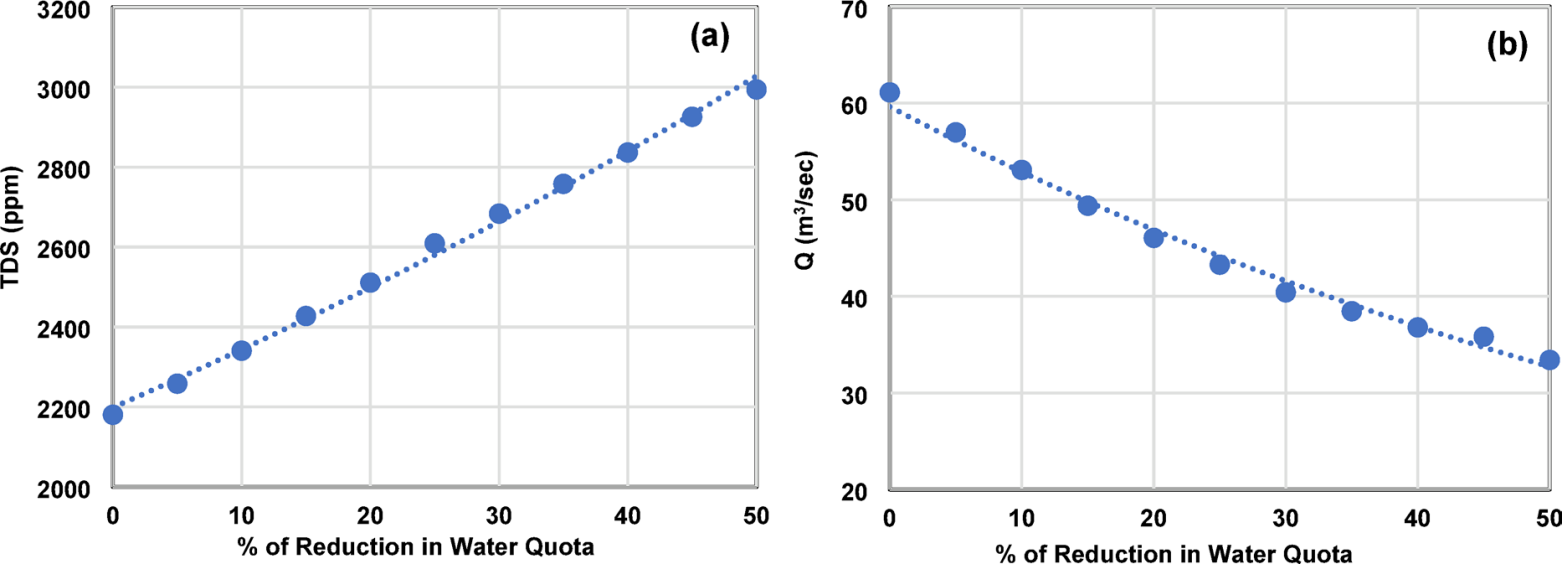
(**a**) TDS concentration (ppm) versus water quota reduction at BBF; (**b**) Discharge (m³/s) versus water quota reduction at BBF.

At the BBF site, baseline TDS reaches approximately 2200 ppm, already exceeding the 2000 ppm limit under Egyptian Law 48/1982^24^. Under a 50% reduction scenario, TDS at BBF exceeds 3000 ppm (Fig. 6a). This represents a 36% increase from baseline, accompanied by a 47% decline in discharge (Fig. 6b). Both relationships follow exponential functions with R² ≈ 0.98, described by Eqs. 5 and 6, where x represents the percentage reduction in freshwater allocation relative to baseline. The associated 95% confidence intervals (± 5% for TDS, ± 6% for discharge) reflect the scatter of simulated data points around the fitted curve and quantify the precision of empirical relationships.

5 $$\:{TDS}_{BBF}=2198.7\times\:{e}^{0.006x}$$

6 $$\:{Q}_{BBF}=59.62\times\:{e}^{-0.01x}$$

Based on Eq. 5, restoring compliance would theoretically require a 15.8% increase in Eastern Delta freshwater allocation. However, this value is a diagnostic metric, not a feasible policy option, because Egypt’s total Nile allocation is fixed at 55.5 BCM annually and already under stress.

Three mechanisms explain the observed salinity increases at BBF under reduced allocations. First, diminished freshwater inflows reduce dilution capacity. Second, lower discharge decreases hydraulic flushing efficiency, increasing water residence time and salt accumulation. Third, freshwater scarcity intensifies informal drainage water reuse at upstream calculation units, recycling salts within the basin and amplifying salinity at downstream convergence points such as BBF.

Elevated TDS at BBF under high reduction scenarios exceeds tolerance thresholds for several moderately sensitive crops cultivated in the Eastern Delta and violates Law 48/1982 reuse standards. This renders BBF water unsuitable for agricultural reuse under severe scarcity, placing additional pressure on the Bahr El-Baqar wastewater treatment plant.

**Fig. 7:**
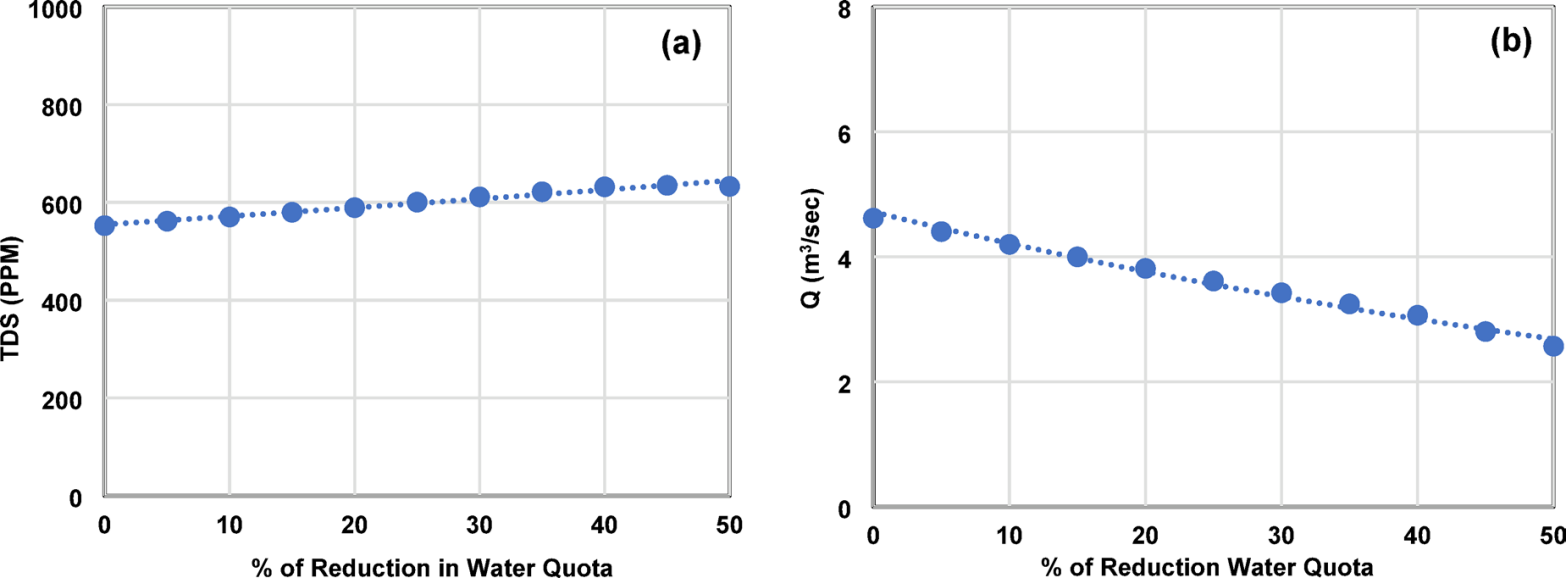
(**a**) TDS concentration (ppm) versus water quota reduction at BAP; (**b**) Discharge (m³/s) versus water quota reduction at BAP.

In contrast, BAP demonstrates greater resilience. TDS remains below 2000 ppm across all scenarios, increasing by only 16.4% under a 50% reduction, from approximately 550 ppm at baseline to 640 ppm (Fig. 7a). Discharge declines by 45% under the same scenario (Fig. 7b). Equations 7 and 8 describe these exponential relationships (R² ≈ 0.98), with 95% confidence intervals of ± 4% for TDS and ± 6% for discharge.

7 $$\:{TDS}_{BAP}=554.73\:\times\:\:{e}^{0.003x}$$

8 $$\:{Q}_{BAP}=4.72\times\:{e}^{-0.011x}$$

### Policy and management implications

This technical target instead quantifies the magnitude of the salinity challenge at BBF and underscores the need for targeted water quality interventions. Given the 3000 ppm TDS exceedance and 47% discharge decline under severe scarcity, a portfolio of deployable interventions that Egyptian agencies can realistically implement includes:

1. Pretreatment with membrane technologies. Hafez et al.^36^ achieved 82% TDS reduction using nanofiltration and 98% monovalent ion removal using reverse osmosis for El-Salaam canal water. Elbialy et al.^37^ confirmed that reverse osmosis (RO) is superior to nanofiltration (NF) for treating agricultural drainage in the Nile Delta. At BBF specifically, where baseline TDS (~ 2200 ppm) already exceeds the 2000 ppm limit, pretreatment is required regardless of freshwater availability.
2. Temporal management of abstraction. Seasonal variability in water quality offers an operational window for optimizing reuse. Long-term monitoring in the northern Nile Delta reveals significant seasonal differences in EC, Na, and Cl between summer and winter in both irrigation and drainage canals^38^. Utilizing BBF water during periods of natural dilution (e.g., winter flows) could reduce TDS concentration without additional treatment.
3. Operational blending optimization. At nodes where source water remains below regulatory limits (e.g., BAP), real-time optimization of mixing ratios based on drainage water quality offers an operational tool for maximizing reuse while maintaining compliance. For BBF, however, blending alone cannot achieve compliance given baseline exceedance.
4. Crop pattern adaptation by salinity tolerance. For command areas that must use higher-salinity water, shifting to salt-tolerant crop varieties provides a practical adaptation pathway. El Demerdash et al.^39^ demonstrated that quinoa maintains productivity as an alternative to wheat under high-salinity irrigation.
5. Upstream salinity source control. Targeted drain cleanup and source control measures can reduce salt loads entering the drainage system before they reach BBF. Abd-Elaty et al.^40^ demonstrated that strategic placement of wastewater treatment plants at sub-basin outlets combined with High-Density Polyethylene (HDPE) stream lining achieves 75.67% TDS reduction in Egyptian drainage systems.
6. Groundwater–drainage interactions management. Managed aquifer recharge and strategic groundwater extraction could influence salinity dynamics at BBF by altering the balance between surface water and groundwater contributions.

These interventions collectively form a deployable portfolio tailored to Egyptian agencies’ capabilities. Under severe scarcity (TDS > 3000 ppm, 47% discharge decline), operational risks include exponentially increased membrane fouling and scaling, higher energy requirements due to elevated osmotic pressure, and shortened maintenance cycles – all directly increasing treatment costs and potentially compromising plant reliability. These penalties underscore the urgency of implementing upstream source control and operational optimization measures to reduce the treatment burden at the plant.

### Comparison with previous studies

The scenario-based projections were compared with prior research on the Eastern Nile Delta. The general direction of simulated trends aligns with Shaban (2020)^32^, who documented long-term increases in both drainage water reuse discharges and salinity. However, while Shaban employed historical trend analysis and probabilistic simulations of reuse expansion, the present study introduces a direct scenario-driven framework that links discharge and salinity responses at specific reuse points (BBF and BAP) to predefined freshwater allocation cuts. This approach captures nonlinear system behaviors under severe scarcity, a dimension less emphasized in trend-based projections.

The findings also correspond with Abdul-Muttalib et al. (2025)^31^, a study that applied SIWARE to assess water reduction impacts on crop productivity and soil salinity across Eastern Delta governorates. Their identification of a stability threshold followed by sharp deterioration under a 50% reduction aligns with the threshold-type responses observed at BBF. Whereas their analysis focused on regional agricultural impacts, the present study provides a node-based perspective that reveals how basin-wide shortages concentrate at strategic control points, offering a more precise tool for infrastructure planning and reuse management.

### Model uncertainties and interpretation of findings

As with all hydrological models, SIWARE simplifies reality, and its projections are subject to uncertainties. These arise from five sources. (1) Input data uncertainty, particularly for future climate conditions and upstream water allocations. (2) Parameter and calibration uncertainty, where estimated values for soil properties and drainage processes have plausible ranges. (3) Structural uncertainty, such as the model’s deterministic representation of informal reuse decisions, which may not capture all behavioral nuances. (4) Conversion uncertainty from EC to TDS, the standard factor 640 is appropriate for Nile Delta freshwater but may introduce uncertainty for drainage water with different ionic composition. Varying the conversion factor from 550 to 750 changes absolute TDS values by ± 15% but does not alter the comparative conclusions about BBF versus BAP resilience. (5) The model assumes fixed crop distribution and cultivated areas across all scenarios. Under severe freshwater reductions, farmers would likely shift to more salt‑tolerant or lower‑water‑demand crops, meaning our projections could overestimate water quality deterioration.

Despite these limitations, the model’s ability to reproduce historical conditions provides a reasonable empirical foundation for scenario analysis. Section  3.1–3.3 demonstrate good agreement with observed data (R² up to 0.92, NSE up to 0.91, Av.dev. within 12.4% for discharge and 20.6% for salinity), indicating that SIWARE adequately simulates baseline discharge and salinity dynamics for the study period.

The 95% confidence intervals (± 4–6%) accompanying the predictive equations (Sect.  4.1) quantify the precision of the exponential relationships and confirm the empirical fits are statistically robust. When interpreting these intervals, it is useful to recognize that they primarily reflect the goodness-of-fit of the regression, while the broader uncertainty context, including input, parameter, and structural uncertainties, is addressed qualitatively in this section.

The core findings, notably that BBF baseline TDS already exceeds the limit, are consistent across scenarios and robust to the qualitative uncertainties discussed above. The finding that BBF baseline TDS already exceeds the regulatory limit is a direct model output, not an extrapolation, and remains robust across the range of conditions considered.

Therefore, model outputs are best interpreted as robust comparative projections across defined scenarios, highlighting relative trends and system vulnerabilities. This framework allows water managers to apply the predictive equations as practical screening tools while remaining aware of the broader uncertainty context discussed above. In addition to these parametric uncertainties, a separate sensitivity analysis was conducted to test how the results respond to alternative compliance scenarios (Sect.  4.5).

### Sensitivity analysis of compliance-based mixing

Since mixing ratios at BBF and BAP are determined by the 2000 ppm TDS compliance standard rather than fixed operational values, a sensitivity analysis was conducted to assess how results would change under alternative compliance scenarios. Two sensitivity tests were performed:

#### Alternative regulatory standards

The 2000 ppm limit was varied to 1500 ppm (stricter) and 2500 ppm (more lenient) under the 50% reduction scenario. At BBF, where baseline TDS already exceeds 2000 ppm, even the more lenient 2500 ppm standard does not restore compliance, as TDS under 50% reduction exceeds 3000 ppm. At BAP, the stricter 1500 ppm standard would still be met under all scenarios, as maximum projected TDS (640 ppm) remains well below this threshold. This indicates that BBF’s compliance failure is robust to reasonable variations in the regulatory standard.

#### Intentional deviation from standards under extreme scarcity

While legally required to maintain TDS ≤ 2000 ppm under current Egyptian law, this sensitivity test explores the technical consequences of relaxing the standard during extreme scarcity events, a hypothetical scenario that would require regulatory change. To test whether managers could temporarily accept higher TDS during severe scarcity, the compliance threshold was relaxed to 3000 ppm under the 50% reduction scenario. At BBF, this would allow continued reuse, but at the cost of irrigating with water exceeding crop tolerance thresholds for sensitive crops. At BAP, even the 2000 ppm standard remains achievable. This suggests that while regulatory flexibility could maintain water availability at BBF during extreme events, it would come with significant agronomic trade-offs.

#### Implications for water management

These sensitivity tests reveal two key insights:

1. At compliance-exceeding nodes (BBF): No realistic adjustment to mixing ratios or regulatory standards can restore compliance without compromising crop suitability. Remediation requires either source water quality improvement (e.g., pretreatment) or substantial additional freshwater beyond what is needed for dilution.
2. At compliant nodes (BAP): The system is robust to both stricter standards and scarcity conditions. Management focus should remain on maintaining freshwater allocations to preserve reusable volumes rather than on quality concerns.

## Conclusions

This study applied the SIWARE model to project drainage water quantity and quality under scarcity at two strategic reuse nodes in the Bahr El-Baqar catchment, Egypt: the Bahr El-Baqar Feeder (BBF) and the Bilad El-Ayad Pump Station (BAP). The analysis produced three main findings.

First, the simulated responses followed exponential relationships (R² ≈ 0.98 for curve fitting), predicting both discharge and salinity at the two sites for any freshwater allocation reduction from 5% to 50%. The associated 95% confidence intervals (± 4–6%) confirm the statistical reliability of these predictive equations for comparative scenario analysis, providing water managers with a direct tool for rapid assessment without requiring full model re-runs.

Second, it identified BBF as a critical compliance hot-spot where baseline salinity (~ 2200 ppm) already exceeds Egypt’s 2000 ppm regulatory limit. Under a 50% allocation cut, salinity escalates to over 3000 ppm, a 36% increase, accompanied by a 47% decline in discharge. This finding underscores the severity of the salinity challenge at BBF. The 15.8% theoretical dilution requirement is unattainable under Egypt’s fixed Nile allocation and rising population pressures, reinforcing that technological or management interventions are necessary. The results flag significant operational risks for the world’s largest treatment plant, including accelerated membrane fouling, increased energy consumption, and shortened maintenance cycles under severe scarcity.

Third, it demonstrated that scarcity impacts are highly node-specific. A 50% reduction caused a 36% salinity increase and 47% discharge decline at BBF, while the more resilient BAP site experienced only a 16.4% salinity increase with a 45% discharge decline. This contrast underscores the necessity of targeted management over regional blanket policies.

Building on these findings, the study presents a deployable portfolio of interventions tailored to Egyptian agencies’ capabilities:

- Pretreatment with membrane technologies, supported by demonstrated TDS reductions (82%) and RO superiority in the Nile Delta.
- Temporal management of abstraction leveraging seasonal water quality variations.
- Operational blending optimization at compliant nodes, with recognition that blending alone cannot restore compliance at BBF.
- Crop pattern adaptation using salt-tolerant varieties such as quinoa.
- Upstream salinity source control achieving up to 75.67% TDS reduction through strategic WWTP placement and HDPE stream lining.
- Groundwater–drainage interactions management to influence salinity dynamics.

The 0–50% reduction scenarios provide quantitative evidence base for adaptive water management, while the node-specific predictive equations enable rapid assessment of both water availability and quality under different allocation policies. The identification of BBF as a compliance hot-spot signals urgent need for prioritized investment in pretreatment technologies and upstream source control measures.

Unlike previous studies that examined regional agricultural impacts (Abdul-Muttalib et al., 2025) or historical trends (Shaban, 2020), this study provides quantitative, node-specific predictions for two strategic reuse points under defined scarcity scenarios ranging from 5 to 50% allocation reductions. The study quantifies trade-offs between water allocation and reuse viability, offering actionable insights for sustaining ADW reuse, a vital unconventional resource under increasing scarcity. This work contributes directly to Sustainable Development Goal (SDG) 6 (clean water and sanitation) through improved water use efficiency and integrated resource management, and to SDG 2 (zero hunger) by informing strategies to safeguard water for food production in water-stressed regions.

## Data Availability

The data can be provided upon request from corresponding author.
